# Comparison of 4 ml of 1% Lignocaine Versus 2 ml of 2% Lignocaine for Landmark-Guided Superior Laryngeal Nerve Block During Awake Fiberoptic Intubation

**DOI:** 10.7759/cureus.113049

**Published:** 2026-07-20

**Authors:** Harish Kumar, Patil S Manoj, Zainab Ahmad, Kiran Molli, Shikha Jain, Akshaya Sreekumar, Vaishali Waindeskar

**Affiliations:** 1 Anesthesiology, All India Institute of Medical Sciences, Bhopal, Bhopal, IND

**Keywords:** airway management, awake fiberoptic intubation, lignocaine, local anesthesia, superior laryngeal nerve block

## Abstract

Background: Superior laryngeal nerve block is one option for airway topicalization during awake fiberoptic intubation. The optimal balance between injectate volume and lignocaine (lidocaine) concentration remains uncertain.

Objective: To compare a larger-volume, lower-concentration regimen (4 mL of 1% lignocaine per side) with a smaller-volume, higher-concentration regimen (2 mL of 2% lignocaine per side). We hypothesized that the larger volume would improve neural spread, increase the proportion of completely relaxed vocal cords, and shorten fiberoptic intubation time without increasing adverse events.

Methods: In this single-center randomized assessor-blinded trial, 72 adults requiring awake fiberoptic nasotracheal intubation were allocated 1:1 to the 1% group or the 2% group (36 per group). Each regimen delivered 40 mg per side. The primary outcome was completely relaxed vocal cords (Grade 1) during bronchoscope negotiation. Secondary outcomes included fiberoptic intubation time, discomfort assessed 24 hours after extubation, serial hemodynamic variables, oxygen saturation, and bleeding. Categorical outcomes were re-analyzed with exact tests. Fiberoptic intubation time was additionally examined in a post hoc multivariable linear model adjusted for age, sex, and body weight.

Results: Grade 1 vocal-cord status occurred in 32/36 (88.9%) patients in the 1% group and 23/36 (63.9%) in the 2% group (odds ratio 4.52, 95% CI 1.31-15.66; Fisher exact P=0.025). Mean fiberoptic intubation time was 129.3 ± 39.0 seconds versus 161.2 ± 56.8 seconds, respectively (mean difference -31.9 seconds, 95% CI -54.9 to -9.0; P=0.007). The adjusted difference was -29.3 seconds (95% CI -52.9 to -5.6; P=0.015). No discomfort at 24 hours was reported by 32/36 (88.9%) versus 24/36 (66.7%) patients (P=0.045). Bleeding occurred in 2/36 (5.6%) versus 7/36 (19.4%) patients (P=0.151). Serial hemodynamic values were broadly similar; an isolated difference in diastolic pressure at one minute was not interpreted as a sustained treatment effect.

Conclusion: For landmark-guided superior laryngeal nerve block, 4 mL of 1% lignocaine per side was associated with more completely relaxed vocal cords and shorter fiberoptic intubation time than 2 mL of 2% lignocaine per side. The findings support further multicenter confirmation using validated airway-anesthesia scales and fully blinded injectate preparation where feasible.

## Introduction

Awake tracheal intubation is recommended when loss of spontaneous ventilation or airway tone after induction of general anesthesia could create unacceptable risk. Flexible bronchoscopy remains an established method, while awake videolaryngoscopy is an evidence-supported alternative in appropriately selected patients. Successful awake intubation depends on oxygenation, patient cooperation, judicious sedation, effective airway topicalization, and operator expertise [1-4].

Airway topicalization can be achieved by nebulization, atomization, spray-as-you-go application, translaryngeal injection, or regional nerve blocks [1,5,6]. A bilateral superior laryngeal nerve block is therefore an optional component rather than an obligatory step. The internal branch of the superior laryngeal nerve supplies dense sensory innervation to the supraglottis, epiglottis, aryepiglottic folds, and laryngeal mucosa above the vocal cords; blockade may reduce glottic closure, coughing, and discomfort during scope passage [7,8]. Comparative trials and evidence syntheses suggest that airway nerve blocks can shorten intubation time and improve intubating conditions compared with topicalization without nerve blocks, although techniques and local-anesthetic regimens are heterogeneous [9,10].

Lignocaine is reported in multiple concentrations and volumes for superior laryngeal nerve block. At an equal drug mass, a larger volume may theoretically improve spread within the thyrohyoid region, whereas a higher concentration may produce a steeper local concentration gradient. Direct clinical comparisons of equipotent regimens that isolate this volume-concentration trade-off are limited. Landmark-guided injection is particularly relevant where ultrasound is unavailable, but precise localization may be less consistent than with ultrasound guidance, making injectate spread potentially important [8,11-13].

The primary objective was to compare the proportion of completely relaxed vocal cords during bronchoscope negotiation after 4 mL of 1% lignocaine versus 2 mL of 2% lignocaine per side. Secondary objectives were to compare fiberoptic intubation time, need for supplemental spray-as-you-go lignocaine, serial hemodynamic variables, oxygen saturation, post-procedure discomfort, and bleeding. The superiority hypothesis was that the larger-volume 1% regimen would produce better vocal-cord relaxation and shorter intubation time because of wider local spread, while maintaining the same total lignocaine dose.

## Materials and methods

Study design, setting, and ethics

This prospective, single-center, randomized, parallel-group trial was conducted in the operating theatres of the Department of Anaesthesiology and Critical Care, All India Institute of Medical Sciences, Bhopal, India, over an 18-month recruitment period. The Institutional Human Ethics Committee approved the protocol (AIIMS/BPL/IECSR/JAN/23/PG/06; August 24, 2023). Written informed consent was obtained before enrollment. The revised report describes the design as assessor-blinded rather than double-blinded because the clinician administering the block could identify the allocated volume.

Participants

Adults aged 18-70 years with American Society of Anesthesiologists physical status I-II who required elective awake fiberoptic bronchoscopy-guided nasotracheal intubation were eligible. Exclusion criteria were hemodynamic instability, coagulopathy, restricted neck extension (<35 degrees as documented in the protocol), allergy to lignocaine or another study drug, a front-of-neck mass that obscured landmarks, epilepsy, pregnancy, known carotid plaque, or recent stroke.

Sample size

The protocol used the proportion of completely relaxed vocal cords reported with 2 mL of 2% lignocaine in the study by Gupta et al. [9]. Assuming an increase from 50% to 80% with 4 mL of 1% lignocaine, 80% power, a two-sided alpha of 0.05, and G*Power version 3.1.9.7 (Heinrich-Heine-Universität Düsseldorf, Düsseldorf, Germany), the target sample was 72 patients (36 per group).

Randomization, allocation concealment, and blinding

A computer-generated random sequence allocated participants in a 1:1 ratio. Allocation was concealed in sealed envelopes. The patient, the anesthesiologist performing fiberoptic intubation, and the observer recording outcomes were unaware of allocation. A separate anesthesiologist opened the envelope and performed the superior laryngeal nerve block. Because the study volumes differed, this block operator was necessarily unblinded and did not participate in intubation or outcome assessment. Clinicians performing the blocks had previously performed at least 20 superior laryngeal nerve blocks; clinicians performing fiberoptic intubation had completed at least 15 awake fiberoptic intubations.

Standardized preparation and intervention

Patients received oral alprazolam 0.25 mg on the night before surgery, intramuscular glycopyrrolate 0.2 mg 30 minutes before intubation, two drops of xylometazoline in each nostril, and nebulization with 4 mL of 4% lignocaine for 15-20 minutes. The nostrils were prepared with a nasopharyngeal airway lubricated with 2% lignocaine gel. Standard electrocardiography, non-invasive blood pressure, pulse oximetry, and capnography were applied. The use of an antisialagogue, nasal vasoconstrictor, topical local anesthetic, continuous monitoring, and cautious sedation is consistent with contemporary awake-intubation recommendations [1,5,6].

After identification of the hyoid bone and thyrohyoid membrane, the 1% group received 4 mL of 1% lignocaine on each side (2 mL deposited after piercing the thyrohyoid membrane and 2 mL superficial to it). The 2% group received 2 mL of 2% lignocaine on each side (1 mL deep and 1 mL superficial to the membrane). Both regimens, therefore, delivered 40 mg per side and 80 mg in total for bilateral blockade. The target anatomy and landmark approach were consistent with published anatomical and clinical descriptions of superior laryngeal nerve block [8,11]. A translaryngeal injection of 4 mL of 2% lignocaine was administered in both groups. The original protocol did not specify a five-minute interval between the two sides; that statement in the previous manuscript has been removed. At least 10 minutes elapsed after completion of the airway blocks before fiberoptic intubation was attempted.

If the vocal cords were adducted during attempted bronchoscope passage, 1-mL aliquots of 2% lignocaine were applied to the glottis through an epidural catheter placed in the working channel of the bronchoscope. At least 30 seconds were allowed between aliquots. Total lignocaine exposure was maintained below 9 mg/kg, with the cumulative dose accounting for nebulized, gel, block, translaryngeal, and spray-as-you-go administration [1,14,15]. Dexmedetomidine 0.5 micrograms/kg was infused over 10 minutes in both groups; randomized studies have supported dexmedetomidine as a cooperative-sedation option for awake fiberoptic intubation [16,17]. A 6.5-mm flexometallic nasotracheal tube was used in female patients and a 7.0-mm tube in male patients.

Outcome definitions and measurement

The primary outcome was vocal-cord adduction during attempted bronchoscope negotiation. The original case-record form used an investigator-defined three-grade ordinal scale: Grade 1 denoted completely relaxed cords, while Grades 2 and 3 denoted progressively greater adduction. The scale was not a validated named instrument, and the source record did not preserve a more granular operational distinction between Grades 2 and 3. The primary comparison was therefore prespecified for this revision as Grade 1 versus Grades 2-3, with the full three-level distribution also reported.

Fiberoptic intubation time was defined as the elapsed time from insertion of the bronchoscope into the nostril until the first end-tidal carbon dioxide waveform was observed after tracheal-tube placement. A blinded observer started and stopped the timing device and recorded the value in seconds; the device model was not retained in the source record. Patient discomfort was assessed 24 hours after extubation as no, mild, or severe discomfort. A 10-point visual analog comfort score was not part of the original case-record form and has been removed from the revised manuscript. Heart rate, systolic and diastolic blood pressure, and oxygen saturation were summarized at 1, 3, 6, 9, and 10 minutes. Bleeding was recorded as present or absent.

Statistical analysis

The participant-level master chart was reconciled against the thesis record before analysis. Continuous variables are reported as mean ± standard deviation and compared with Welch independent-samples t tests. Categorical 2 x 2 outcomes were compared with two-sided Fisher exact tests. For the full three-category vocal-cord and discomfort distributions, a Fisher-Freeman-Halton exact test was estimated by Monte Carlo resampling; ordinal Mann-Whitney U results were used as sensitivity analyses. Effect estimates are presented with 95% confidence intervals where appropriate. Fiberoptic intubation time was additionally analyzed in a post hoc multiple linear regression model including treatment group, age, sex, and body weight, with heteroscedasticity-consistent HC3 standard errors. Mallampati grade was not available in the original case-record form and could not be entered into the model; all participants had adequate neck extension. Serial hemodynamic comparisons are descriptive and pointwise, without multiplicity adjustment. Two-sided P<0.05 was considered statistically significant. Analyses were performed with Python 3 (Python Software Foundation, Fredericksburg, VA, US) using SciPy (NumFOCUS Foundation, Austin, TX, USA) and statsmodels.

## Results

Participant flow and baseline characteristics

**Figure 1:**
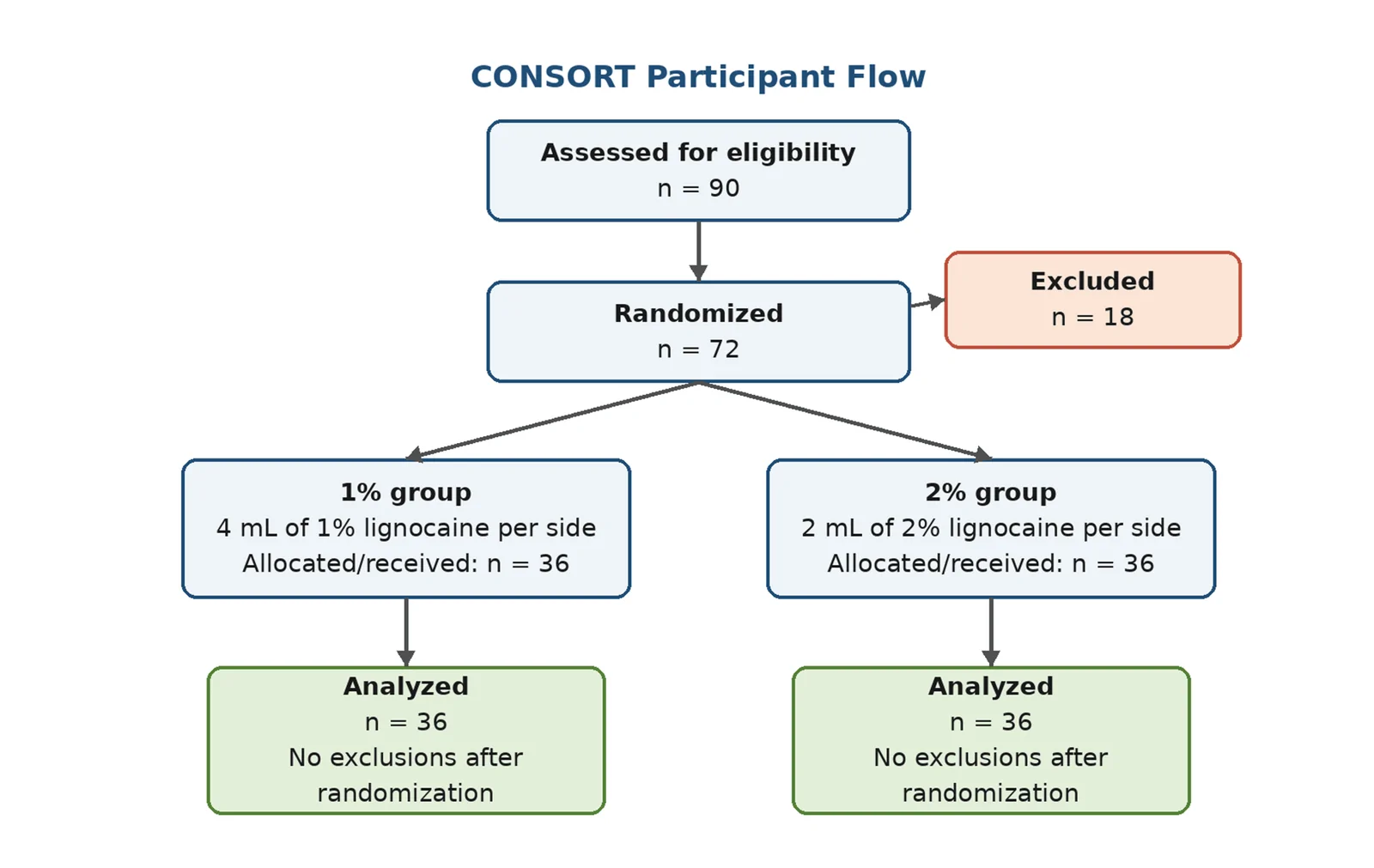
CONSORT participant-flow diagram. CONSORT, Consolidated Standards of Reporting Trials.

Ninety patients were assessed for eligibility, 18 were excluded before randomization, and 72 were randomized. All 72 received the allocated intervention and were included in the analysis: 36 in the 1% group and 36 in the 2% group (Figure 1).

No post-randomization loss or exclusion was documented.

The groups were similar in age, body weight, body mass index, sex distribution, and neck extension (Table 1).

**Table 1:** Baseline characteristics. Continuous variables: Welch independent-samples t test; sex: two-sided Fisher exact test.

Characteristic	1% group (n=36)	2% group (n=36)	Test statistic	P value
Age, years	49.0 ± 10.4	49.1 ± 9.4	t=-0.04	0.972
Male sex, n (%)	17 (47.2)	22 (61.1)	Fisher exact	0.344
Body weight, kg	62.6 ± 10.1	66.1 ± 12.2	t=-1.29	0.202
Body mass index, kg/m²	23.1 ± 2.3	23.4 ± 2.9	t=-0.54	0.588
Adequate neck extension, n (%)	36 (100)	36 (100)	Not estimable	—

Vocal-cord adduction

Completely relaxed cords (Grade 1) were observed in 32/36 (88.9%) patients in the 1% group and 23/36 (63.9%) in the 2% group. The odds of Grade 1 status were higher with the 1% regimen (odds ratio 4.52, 95% CI 1.31-15.66; Fisher exact P=0.025). The full distribution is shown in Table 2; the three-category exact comparison yielded P=0.060, while the ordinal sensitivity analysis yielded P=0.013.

**Table 2:** Vocal-cord adduction. Primary exact comparison: Grade 1 versus Grades 2-3, Fisher exact P=0.025. Full 2 × 3 Fisher-Freeman-Halton Monte Carlo P=0.060. The grading system was investigator-defined and not a validated named scale.

Vocal-cord grade	1% group (n=36)	2% group (n=36)
Grade 1: completely relaxed	32 (88.9)	23 (63.9)
Grade 2: intermediate adduction	3 (8.3)	9 (25.0)
Grade 3: greatest adduction	1 (2.8)	4 (11.1)
Grade 1 vs. Grades 2-3	32/36	23/36

Fiberoptic intubation time

Fiberoptic intubation time was shorter in the 1% group (129.3 ± 39.0 seconds) than in the 2% group (161.2 ± 56.8 seconds). The unadjusted mean difference was -31.9 seconds (95% CI -54.9 to -9.0; Welch t=-2.78, P=0.007; Cohen d=-0.66) (Table 3).

**Table 3:** Fiberoptic intubation time. Welch independent-samples t test. Negative values favor the 1% group. FOI, fiberoptic intubation.

Outcome	1% group (n=36)	2% group (n=36)	Effect estimate	P value
FOI time, seconds	129.3 ± 39.0	161.2 ± 56.8	-31.9 s (95% CI -54.9 to -9.0)	0.007

In the post hoc multivariable model adjusted for age, sex, and body weight, allocation to the 1% group remained independently associated with a 29.3-second shorter intubation time (95% CI -52.9 to -5.6; P=0.015; Table 4).

**Table 4:** Post hoc multiple linear regression for FOI time with HC3 robust standard errors (n=72; R²=0.134; adjusted R²=0.082). A negative coefficient indicates a shorter time. FOI, fiberoptic intubation; HC, heteroskedasticity-consistent.

Predictor	Adjusted coefficient, s	95% CI	P value
1% group (reference: 2% group)	-29.3	-52.9 to -5.6	0.015
Age, per year	0.5	-0.6 to 1.7	0.366
Male sex	7.8	-20.1 to 35.7	0.584
Body weight, per kg	0.4	-0.6 to 1.5	0.390

Discomfort, hemodynamics, and bleeding

At 24 hours after extubation, no discomfort was reported by 32/36 (88.9%) patients in the 1% group and 24/36 (66.7%) in the 2% group (odds ratio 4.00, 95% CI 1.15-13.95; Fisher exact P=0.045). The full three-category exact comparison yielded P=0.113; the ordinal sensitivity analysis yielded P=0.024 (Table 5).

**Table 5:** Post-extubation discomfort. Binary comparison (no discomfort versus any discomfort): Fisher exact P=0.045. Full 2 × 3 Fisher-Freeman-Halton Monte Carlo P=0.113.

Discomfort at 24 hours	1% group (n=36)	2% group (n=36)
No discomfort	32 (88.9)	24 (66.7)
Mild discomfort	3 (8.3)	8 (22.2)
Severe discomfort	1 (2.8)	4 (11.1)

Both groups maintained stable hemodynamic parameters throughout the procedure. No significant inter-group differences were observed in heart rate, systolic blood pressure (SBP), or diastolic blood pressure (DBP) at any time point (Table 6).

**Table 6:** Serial hemodynamic measurements. Values are mean ± SD. P values are pointwise Welch tests and were not adjusted for repeated testing. The isolated 1-minute diastolic-pressure difference (P=0.034) was not sustained. Oxygen satururation (SpO₂) was 100% in both groups at all recorded time points.

Variable	Minute	1% group	2% group	P value
Heart rate, beats/min	1	79.8 ± 12.0	84.2 ± 15.2	0.178
	3	82.5 ± 10.3	85.9 ± 13.4	0.230
	6	80.9 ± 10.0	84.8 ± 12.6	0.155
	9	80.4 ± 9.5	84.4 ± 12.1	0.126
	10	80.1 ± 9.3	83.8 ± 11.8	0.140
Systolic blood pressure, mmHg	1	118.9 ± 11.7	124.2 ± 11.4	0.052
	3	119.7 ± 11.3	124.0 ± 11.1	0.113
	6	119.2 ± 10.7	123.2 ± 9.9	0.099
	9	118.5 ± 9.6	122.4 ± 9.6	0.088
	10	118.3 ± 8.9	122.1 ± 9.2	0.078
Diastolic blood pressure, mmHg	1	74.6 ± 8.4	78.9 ± 8.5	0.034
	3	75.1 ± 7.4	77.4 ± 9.6	0.258
	6	74.5 ± 7.5	76.0 ± 8.5	0.428
	9	74.3 ± 7.4	75.7 ± 8.2	0.444
	10	74.4 ± 7.1	75.4 ± 8.2	0.571

Bleeding was recorded in 2/36 (5.6%) patients in the 1% group and 7/36 (19.4%) in the 2% group. The difference did not reach statistical significance after exact re-analysis (odds ratio for 2% versus 1% 4.10, 95% CI 0.79-21.32; Fisher exact P=0.151). No claim of a confirmed reduction in bleeding is therefore made.

## Discussion

This randomized assessor-blinded trial found that a larger-volume, lower-concentration lignocaine regimen produced a higher proportion of completely relaxed vocal cords and shorter fiberoptic intubation time than an equipotent smaller-volume, higher-concentration regimen. The time difference remained after post hoc adjustment for age, sex, and body weight. Post-extubation discomfort also favored the 1% regimen, although the discomfort scale was investigator-defined and the study was not powered for safety outcomes.

The findings are biologically plausible. Both regimens delivered the same lignocaine mass, but 4 mL may distribute more widely across the thyrohyoid region under landmark guidance, potentially reaching more branches of the internal superior laryngeal nerve [7,8]. This mechanistic explanation remains inferential because injectate spread was not imaged. The result should not be interpreted as evidence that a superior laryngeal nerve block is mandatory for every awake intubation. Current guidance and reviews emphasize a range of topicalization techniques tailored to the patient, operator, and local resources [1,2,5,6]. Rather, the study addresses the narrower question of how two commonly feasible block regimens perform when this block is selected.

Our results are consistent with the broader literature suggesting that regional airway blocks can improve intubating conditions. Gupta et al. reported shorter intubation time and better conditions with airway nerve blocks than with nebulized lignocaine in patients with cervical-spine injury [9]. A 2023 meta-analysis of 14 randomized trials found that airway nerve blocks reduced intubation time and cough or gag responses and improved patient satisfaction compared with topicalization without nerve blocks [10]. Ambi et al. demonstrated that ultrasound guidance improved airway-anesthesia quality and reduced intubation time compared with a landmark approach in their setting, whereas Magadum et al. reported that landmark-guided block can still provide clinically acceptable conditions in experienced hands [11,12]. Wang et al. found that adding ultrasound-guided internal-branch block to a standardized regimen shortened intubation time in patients with severe chronic obstructive pulmonary disease [13]. Khandelwal et al. reported improved anesthesia quality when nebulized lignocaine was added to airway blocks [18]. Mohanta et al. used 1 mL of 2% lignocaine per side for ultrasound-guided superior laryngeal nerve block and observed shorter intubation time than with ultrasonic nebulization [19]. Kundra et al. also demonstrated successful awake nasotracheal intubation using different local-anesthesia strategies, emphasizing the long-standing heterogeneity of protocols [20].

The exact re-analysis materially changes several interpretations from the earlier manuscript. The Grade 1 vocal-cord endpoint remained statistically significant when analyzed as the binary endpoint used in the sample-size rationale. In contrast, the apparent bleeding advantage did not remain statistically significant with Fisher exact testing. The revised conclusion therefore focuses on vocal-cord relaxation and intubation time rather than claiming fewer complications. Similarly, the unsupported VAS comfort scores, procedural-success percentages, coughing and gagging rates, and “excellent-to-good” intubation-condition categories have been removed because they were not present in the original case-record form or master chart.

The hemodynamic table shows no consistent group separation. One isolated difference in diastolic pressure at one minute was observed, but it was one of multiple pointwise comparisons and was not sustained. This pattern does not support a clinically important hemodynamic effect of either regimen. Rare severe hypotension and bradycardia after superior laryngeal nerve block have nevertheless been reported, supporting continued monitoring despite stable group means in the present trial [21]. A recent randomized trial comparing two ultrasound-guided superior laryngeal nerve block approaches also found that the block approach can influence airway-anesthesia quality and procedure time, underscoring that technique may interact with volume and concentration [22]. Both groups received identical co-interventions, including nebulized and translaryngeal lignocaine and dexmedetomidine, which reduces confounding between groups but also means that the findings apply to the complete airway-anesthesia bundle rather than to an isolated superior laryngeal nerve block.

Strengths and limitations

Strengths include randomized allocation, concealed assignment, separation of the unblinded block operator from the blinded intubator and observer, use of equal lignocaine mass in both regimens, complete follow-up, reconciliation with the participant-level master chart, exact re-analysis of categorical outcomes, effect estimates with confidence intervals, and a covariate-adjusted sensitivity analysis for intubation time.

Several limitations require emphasis. First, the block operator could not be blinded because of the visible volume difference, creating potential performance bias; the design is therefore assessor-blinded rather than double-blinded. Second, the vocal-cord and discomfort scales were investigator-defined and not validated, and the source record did not retain a detailed operational distinction between Grades 2 and 3. Third, the trial was single-center and included a modest sample, limiting precision for uncommon adverse events and external validity. Fourth, Mallampati grade and other detailed airway-anatomy measures were not available in the original case-record form for covariate adjustment; all participants had adequate neck extension. Fifth, serial hemodynamic analyses were descriptive and not modeled longitudinally. Sixth, plasma lignocaine concentrations and supplemental spray-as-you-go dose by participant were not available for the revised analysis. Finally, the use of standardized nebulized and translaryngeal lignocaine means the incremental contribution of the superior laryngeal nerve block cannot be separated from the full topicalization protocol.

## Conclusions

In adults undergoing awake fiberoptic nasotracheal intubation with a standardized airway-anesthesia bundle, landmark-guided superior laryngeal nerve block with 4 mL of 1% lignocaine per side was associated with more completely relaxed vocal cords and a clinically relevant reduction in intubation time compared with 2 mL of 2% lignocaine per side. The larger-volume regimen may be considered when landmark guidance is used, but the result should be confirmed in multicenter trials using validated outcome scales, detailed airway covariates, and a blinding strategy that minimizes operator bias.
